# Feasibility of DNA Methylation Age as a Biomarker of Symptoms and Resilience among Cancer Survivors with Multiple Chronic Conditions

**DOI:** 10.3390/biomedicines11113076

**Published:** 2023-11-16

**Authors:** Nada Lukkahatai, Jongmin Park, Hejingzi Monica Jia, Daniel Martin, Junxin Li, Jennifer Yeong-Shin Sheng, Jessica Gill, Leorey N. Saligan, Vered Stearns, Michael Carducci

**Affiliations:** 1 School of Nursing, Johns Hopkins University, Baltimore, MD 21205, USA; dmart130@jh.edu (D.M.); junxin.li@jhu.edu (J.L.); jessicagill@jhu.edu (J.G.); 2 College of Nursing, Research Institute of Nursing Science, Pusan National University, Yangsan-si 50612, Republic of Korea; jmpark@pusan.ac.kr; 3 Bloomberg School of Public Health, Johns Hopkins University, Baltimore, MD 21205, USA; hjia11@jhu.edu; 4 Johns Hopkins School of Medicine, Sidney Kimmel Comprehensive Cancer Center, Baltimore, MD 21205, USA; jsheng7@jhmi.edu; 5 National Institute of Nursing Research, National Institutes of Health, Bethesda, MD 20892, USA; saliganl@mail.nih.gov; 6 School of Medicine, Johns Hopkins University, Baltimore, MD 21205, USAcarducci@jhmi.edu (M.C.)

**Keywords:** DNA methylation age, biomarkers, symptom, resilience, cancer survivor

## Abstract

This study aims to examine the feasibility of DNA methylation age as a biomarker for symptoms and resilience in cancer survivors with multiple chronic conditions (MCCs). We included ten participants from our parent study, an ongoing randomized control trial study. Participants’ symptoms and resilience were assessed, and peripheral blood was collected. DNA methylation age calculation was performed using DNAge^®^ analysis. Data were analyzed using Spearman’s correlation analysis and the Mann–Whitney U test. Participants in the intervention group tended to have a decrease in DNA methylation age and age acceleration after completing an exercise program (mean difference = −0.83 ± 1.26). The change in DNA methylation age was significantly correlated with the change in resilience score (r = −0.897, *p* = 0.015). The preliminary results suggest that DNA methylation age can be a potential biomarker for improving resilience in cancer survivors with multiple chronic conditions. This finding is limited by the small sample size, and a larger study is needed.

## 1. Introduction

Deoxyribonucleic acid (DNA) methylation (DNAm) refers to the addition of a methyl group to DNA molecules, which varies with aging. It has been linked to a spectrum of health conditions. This phenomenon, characterized by an individual’s biological age advancing faster than their chronological age, has been associated with diverse ailments. These include an elevated risk of developing cancer, increased vulnerability to frailty in aging populations, and the potential to disrupt sleep patterns [1,2,3,4,5,6,7,8]. Furthermore, accelerated DNA methylation age has been implicated in the onset and progression of Alzheimer’s disease, as well as cognitive impairment [9]. Notably, it has also been connected to higher mortality rates [10]. The mechanisms underpinning these associations are multifaceted. One pivotal factor is the activation of inflammatory pathways within the body. Accelerated DNA methylation age seems to promote chronic inflammation, which is a well-known contributor to various chronic diseases, including cancer [11]. Additionally, this biological phenomenon may compromise the DNA damage response mechanisms that typically repair genetic mutations. This weakening of DNA repair processes could heighten susceptibility to genetic damage and disease development. Another critical aspect involves the impact of accelerated DNA methylation age on mitochondrial function [12]. Mitochondria, the cellular powerhouses responsible for energy production, appear to exhibit altered behavior in response to shifts in DNA methylation age. These changes in mitochondrial signatures can potentially result in cellular dysfunction, further exacerbating the aging process and contributing to age-related health concerns. 

The DNA methylation age, also referred to as the “epigenetic clock” or “epigenetic age”, is a prominent epigenetic age estimator that serves as a predictive tool for assessing an individual’s biological age, often synonymous with their physiological or phenotypical age. This estimation is achieved through examining alterations in the patterns of DNA methylation within an individual’s DNA molecules [13]. The DNA methylation age has gained significant attention within the field of aging research due to its remarkable efficacy in predicting both chronological age and various health outcomes when compared with other age estimation methods, such as telomere length assessment, transcriptomic-based estimators, and proteomic-based estimators [14]. In essence, the DNA methylation age estimator stands out as a highly promising and accurate means of gauging an individual’s biological age and their potential health status via scrutinizing epigenetic changes in their DNA, offering valuable insights into its implications for overall well-being.

Epigenetic age acceleration may have a significant role in the development, severity, and persistence of various health symptoms such as pain, fatigue, anxiety, and cognitive difficulties, as well as an individual’s resilience to these challenges. For instance, accelerated epigenetic aging may contribute to the earlier onset and increased severity of these symptoms, potentially making individuals more susceptible to health issues. Conversely, those with slower epigenetic aging may exhibit greater resilience, experiencing milder symptoms and better overall health as they age. This intricate interplay between epigenetic age acceleration and health outcomes underscores the importance of understanding and potentially modulating epigenetic processes to promote healthier aging and enhance an individual’s capacity to cope with age-related health challenges. People with positive epigenetic age acceleration (estimated DNA methylation-predicted age older than their chronological age) have high pain sensitivity [15], fatigue [16], age-related cognitive decline [17], and anxiety [18]. Individuals who have negative epigenetic age acceleration reported no chronic pain [15]. Resilience or positive adaptation ability influences the perception of symptoms [19,20,21,22,23,24]. The relationship between high resilience and low self-reported symptoms was well supported [25,26,27,28]. However, little is known about the relationship between resilience and aging. More studies are needed to investigate the relationship between symptoms, resilience, and DNA methylation age.

Individuals with two or more chronic conditions are defined as having multiple chronic conditions (MCCs) [29]. Over 80% of individuals living with cancer have other chronic conditions, including type 2 diabetes, cardiovascular disease, and osteoarthritis [30]. MCCs have become increasingly prevalent in the global population, presenting a complex challenge to both healthcare providers and individuals alike. The management and understanding of MCCs have gained significance in recent years, as they not only impact the symptom burden experienced by affected individuals but also potentially influence their molecular aging processes, as reflected in their DNA methylation age. Cancer survivors with other chronic conditions experience significantly higher symptom burden and severity (e.g., pain and fatigue) than those without any comorbidities [31,32,33,34]. The burden from these symptoms negatively affects their health-related quality of life, resilience, and treatment adherence [28,35,36]. Physical activity is one of the most effective interventions for managing chronic conditions [37]. Continuous moderate-intensity aerobic exercise can improve symptoms and quality of life, delay the onset and progression of chronic conditions [38,39,40,41,42,43], and impact epigenetic modification. Physically active individuals (walking at least 1500 steps/day) have a low epigenetic age [44]. The genome-wide DNA methylation age can be reduced with an 8-week combined diet, exercise, and lifestyle program [45]. However, the association between increasing only physical activity during participation in a tailored home-based exercise program and DNA methylation age is unknown, especially in cancer survivors with MCCs.

In this study, we aim to examine the feasibility of DNA methylation age as a biomarker for symptoms and resilience in cancer survivors with MCCs. To understand the potential role of DNA methylation age, we investigated the changes in DNA methylation age during a tailored home-based exercise program and examined the association of changes in DNA methylation age with changes in symptoms and resilience.

## 2. Methods

### 2.1. Designs

This is a sub-study of the IRB-approved parent study (Johns Hopkins Medicine Institutional Review Board number: IRB00175781). Informed consent was obtained before the study procedures commenced.

### 2.2. Participants

In the sub-study, participants were included in a pilot randomized clinical trial exploring the feasibility and effects of a tailored home-based exercise program on DNA methylation age, symptoms, and resilience in cancer survivors with MCCs. The parent study recruited participants aged 21 years or older, diagnosed with solid tumor cancer with at least one comorbidity (e.g., hypertension, diabetes, etc.), who completed primary cancer treatment three or more months prior. Participants were excluded if, at the time of enrollment, they were receiving primary cancer treatment, had an active infection, or were actively diagnosed with a psychological disorder. We selected 10 participants who completed the exercise program and provided complete questionnaire data and blood samples to measure DNA methylation age. 

### 2.3. Measurements 

In our study, we utilized established instruments to assess participants’ symptoms and well-being as follows.

Pain: To evaluate their experience of pain, we employed the Patient-Reported Outcomes Measurement Information System (PROMIS) Pain Intensity–Short Form 3a V1.0. This concise instrument encompasses three key pain-related parameters: worst pain, average pain, and current pain. Participants rated their pain levels on a scale ranging from 1 (indicating no pain) to 5 (representing very severe pain). The PROMIS Pain Intensity instrument has undergone rigorous conceptual validation and demonstrated commendable reliability, with test-retest values ranging from 0.83 to 0.93 [46].

Fatigue: We assessed participants’ levels of fatigue using the PROMIS Short Form V1.0-Fatigue 6a. This instrument comprises six items designed to capture facets of fatigue, including its frequency, duration, intensity, and impact on physical, mental, and social activities. Respondents provided their ratings on a scale with five response options, ranging from 1 (indicating “never”) to 5 (signifying “always”). The reliability of this fatigue assessment instrument, as determined using Cronbach’s α, was found to be 0.93 [47].

Cognitive function: To evaluate the subjective cognitive function among our study participants, we employed two distinct tools. The Multiple Ability Self-Report Questionnaire (MASQ) featured 38 items aimed at assessing the perceived cognitive difficulties across five domains: language, visual–perceptual ability, verbal memory, visual–spatial memory, and attention/concentration. Participants rated the frequency of these difficulties on a scale from 1 (indicating “Never”) to 5 (representing “Always”). The MASQ exhibited high internal consistency, with a Cronbach’s α coefficient of 0.92 [48]. For the objective measurement of cognitive function, we employed the Montreal Cognitive Assessment (MoCA) to screen for early-stage cognitive decline or mild cognitive impairment. In our study, the Cronbach’s α coefficient for the MoCA instrument among cancer survivors was 0.79 [49]. 

Resilience: we assessed the participants’ resilience using the Connor-Davidson Resilience Scale (CDRS), a self-report rating scale comprising ten items. Participants indicated the degree to which each statement resonated with their experiences, with response options ranging from 0 (indicating “not true at all”) to 4 (signifying “true nearly all the time”). The CDRS demonstrated good internal consistency, with a Cronbach’s α coefficient of 0.83 [50].

### 2.4. DNA Methylation and Methylation Age Calculation

Participants’ blood samples were collected and preserved in EDTA tubes. These specimens were then stored in a controlled environment within a −80 °C freezer until they were ready for batch analysis. Subsequently, the samples were packaged and transported on ice, ensuring their integrity, during overnight shipment to the Zymo Research facility (Irvine, CA, USA). The DNA extraction and the subsequent methylation experiment were executed. Genomic DNA was extracted from the human blood cells utilizing the Quick-DNA™ Miniprep Plus kit, an efficient product provided by Zymo Research (Irvine, CA, USA). To prepare the DNA for methylation analysis, a bisulfite conversion process was conducted. This process involves the transformation of cytosine molecules into uracil, following the protocols outlined in the EZ DNA Methylation-Lightning™ Kit (Zymo Research, Irvine, CA, USA).

The bisulfite-converted DNA was enriched to facilitate sequencing, focusing on over 500 specific gene loci known to be associated with DNA methylation age. From the sequencing data, the precise DNA methylation values were extracted and employed to calculate the DNA age, utilizing Zymo Research’s proprietary DNAge^®^ predictor. Age acceleration, a critical metric in our analysis, was determined as the disparity between the calculated DNA methylation age and the participants’ chronological age. This procedure ensured the accurate assessment of DNA methylation age and age acceleration, allowing us to investigate the intricate relationship between epigenetic aging and symptoms.

### 2.5. Statistical Analysis

All analyses were conducted using SPSS version 25.0 (IBM SPSS Statistics, SPSS, Chicago, IL, USA). The *p*-value of ≤0.05 was considered statistically significant. Data were denoted as mean ± standard deviation, and categorical variables were expressed in numbers. Data were tested for normality and homogeneity, followed by a non-parametric test. The mean differences between the pre- and post-intervention DNA methylation age and symptoms between the two groups were tested using the Mann–Whitney U test. We calculated Spearman’s correlation coefficient to investigate the relationships between DNA methylation age and change in self-reported outcomes before and after the home-based exercise program. 

## 3. Results

### 3.1. Demographic and General Characteristics of Participants

In this sub-study, a total of 10 participants were included, with 4 participants in the control group and 6 participants in the intervention group. The mean chronological age of the control group was approximately 77.75 years, with a standard deviation of 2.22 years. In contrast, the intervention group had a slightly younger mean chronological age of approximately 67.17 years, with a higher standard deviation of 9.70 years. This suggests that there was a notable age difference between the two groups, with the intervention group being, on average, about ten years younger than the control group. To provide a more precise understanding of this age difference, the report notes that 0.1 years is considered equivalent to approximately 1.2 months.

Gender distribution varied between the two groups, with 50% of the control group participants being female, while the intervention group had a higher proportion of females, with 83.3%. Regarding educational backgrounds, half of the control group participants were college graduates. In contrast, the intervention group had a higher percentage of college graduates, with 66.7% of participants having completed a college-level education. Both groups had approximately 50% of participants who had a history of breast or prostate cancer.

The detailed demographic and general characteristics of the participants are summarized in Table 1, which includes additional information such as specific health conditions, medication usage, and any other relevant variables that might be pertinent to the research objectives of the sub-study. 

### 3.2. Associations among DNA Methylation, Symptoms, and Resilience

Table 2 shows the associations between DNA methylation age, symptoms, and resilience changes. The Δ DNA methylation age (Δ age acceleration) was significantly correlated with the Δ resilience score (r = −0.897, *p* = 0.015). None of the other study outcomes showed any significant correlation with DNA methylation age.

### 3.3. Changes to DNA Methylation Age, Symptoms, and Resilience before and after Intervention

There were no statistically significant differences in all measures between the two groups (Table 3). Participants in the intervention group had a slightly decreased DNA methylation age and age acceleration after completing the exercise program (mean difference = −0.83 ± 1.26), but it was not significant. The following also were not significantly decreased after completing the intervention compared with the control group: symptoms, including pain (mean difference = −1.82 ± 11.68), fatigue (mean difference = −5.86 ± 8.00); cognitive decline (mean difference = −2.25 ± 3.30); perceived cognitive difficulty (mean difference = −3.00 ± 17.54); and insomnia (mean difference = −4.00 ± 6.78). Resilience (mean difference = 2.83 ± 6.43) was not significantly increased in those participants who completed the home-based exercise intervention. 

## 4. Discussion

Based on preliminary results, we suggest the feasibility of DNA methylation as a potential biological marker for resilience. Particularly, this is the first report on the associations between DNA methylation age and resilience in cancer patients with MCCs. Resilience in health refers to an individual’s ability to withstand and adapt to challenges, stressors, or adverse circumstances while maintaining or recovering their physical and psychological well-being [51]. This resilience can manifest in different forms, including mental, emotional, and physical resilience, and it plays a crucial role in promoting overall health and well-being through helping individuals navigate and overcome health challenges. Recent studies suggest that a significant relationship between increasing age and a subsequent increase in resilience can be found among people under extreme stress, such as veterans with post-traumatic distress disorder [52], and among those in the general population during COVID-19 [53]. Few studies have reported the relationship between changes in epigenetic age acceleration and resilience. 

In our study, we have identified a noteworthy correlation between low DNA methylation age acceleration and a pronounced high resilience among cancer survivors facing MCCs. This finding hints at the intriguing possibility that the pace at which an individual’s DNA methylation age progresses may have a direct impact on their resilience, a vital attribute for individuals navigating complex health challenges. A recent study revealed that people with a higher level of resilience experienced slower DNA methylation age acceleration [54]. However, given the limitations associated with our modest sample size, it is imperative to exercise caution when interpreting these results. We acknowledge that our findings are preliminary, and as such, we advocate for a more comprehensive investigation involving larger sample sizes. 

The findings from our study provide further validation for the established connections between DNA methylation age and physical activity, reinforcing the outcomes of prior research. Numerous studies have previously suggested that individuals with lower levels of physical activity tend to exhibit an acceleration in DNA methylation age [7,55,56,57]. This link underscores the potential impact of lifestyle choices on the aging process at the epigenetic level. Remarkably, lifestyle modification programs that encompass a holistic approach, combining elements such as dietary adjustments, exercise regimens, improved sleep patterns, relaxation guidance, and nutritional supplementation, have demonstrated the capacity to mitigate the progression of DNA methylation age. Some of these programs have been shown to effectively reduce the DNA methylation age by an impressive range of 1.62 to 1.96 years [45,58]. In our specific study, following a 12-week exercise intervention tailored to individual preferences and physical condition, we observed a reduction in DNA methylation age by approximately 0.83 years among our study participants. This outcome serves as a notable testament to the potential of personalized home-based exercise interventions in influencing DNA methylation age. It emphasizes the significance of targeted lifestyle modifications, particularly exercise, in potentially slowing down the epigenetic aging process. However, it is important to acknowledge that while our results are promising, they also highlight the need for further exploration and investigation to comprehensively understand the intricate relationship between physical activity and DNA methylation age and to optimize tailored interventions for enhancing the epigenetic well-being of individuals.

Recent research has suggested a link between MCCs and alterations in DNA methylation age [59]. Studies have indicated that individuals with MCCs may experience accelerated DNA methylation aging, meaning their cells appear older at the epigenetic level compared with their chronological age. This acceleration in DNA methylation age is thought to be influenced by the chronic inflammatory state often associated with MCCs. Chronic inflammation can trigger epigenetic changes that may accelerate the aging of cells, potentially contributing to age-related health problems [11]. Moreover, MCCs are associated with increased oxidative stress, which can also impact DNA methylation patterns and promote epigenetic aging. Oxidative stress occurs when there is an imbalance between the production of harmful free radicals and the body’s ability to neutralize them. This oxidative stress can damage DNA and the epigenetic marks on it, potentially influencing the rate of DNA methylation aging [60].

Our study has strengths and limitations that are important to acknowledge. A notable challenge we encountered was the disruption caused by the COVID-19 pandemic, which significantly affected our data collection efforts in the parent study. Consequently, we had to work with a considerably small sample size for our analysis. This limitation can impact the generalizability of our findings, as a smaller sample size may not fully capture the diversity and nuances present in a larger population. Furthermore, our study relied exclusively on one method, Horvath’s method, to assess DNA methylation aging. While this method is widely recognized and employed in epigenetic clock research, it is important to note that recent advancements have introduced second-generation clocks like PhenoAge and GrimAge. These newer methods are designed to enhance the precision of biological age estimation and establish stronger associations with clinical outcomes, suggesting that our study could potentially benefit from incorporating these advanced techniques to further refine our understanding of epigenetic aging [54].

The impact of these limitations underscores the need for cautious interpretation of our study’s results. While our findings contribute valuable insights into the relationship between DNA methylation age and various factors, the constraints imposed by our small sample size and the exclusive use of Horvath’s method call for further research that incorporates larger and more diverse participant groups along with updated epigenetic clock methodologies. Such efforts can facilitate a more comprehensive understanding of the intricate links between epigenetic aging and health outcomes, ultimately enhancing the reliability and applicability of our findings in the broader context of health and aging research.

## 5. Conclusions

In summary, our study has shed light on initial findings that underscore the promising role of DNA methylation age as a potential biomarker for enhancing resilience among cancer survivors grappling with MCCs. These preliminary results provide valuable insights into the complex interplay between epigenetic aging and an individual’s ability to withstand and adapt to the myriad challenges posed by both cancer and co-occurring chronic health issues. However, it is essential to approach these findings with caution, given the constraints imposed by our study’s relatively small sample size. To bolster the robustness and generalizability of our observations, we recommend the validation of the relationship between DNA methylation age and symptomatology using a larger and more diverse participant pool in future investigations.

## Figures and Tables

**Table 1:** Demographics and general characteristics of participants.

Variables	Categories	Total (*n* = 10)	Control (*n* = 4)	Intervention (*n* = 6)
Chronological age * (years)		71.40 ± 9.16	77.75 ± 2.22	67.17 ± 9.70
Gender	Male	3 (30.0)	2 (50.0)	1 (16.7)
	Female	7 (70.0)	2 (50.0)	5 (83.3)
Race	White	8 (80.0)	3 (75.0)	5 (83.3)
	Black or African-American	2 (20.0)	1 (25.0)	1 (16.7)
Education	Less than college	4 (40.0)	2 (50.0)	2 (33.3)
	College Graduate	6 (60.0)	2 (50.0)	4 (66.7)
Employment	Employed	2 (20.0)	1 (25.0)	1 (16.7)
	Not employed	8 (80.0)	3 (75.0)	5 (83.3)
Marital status	Married	2 (20.0)	0 (0.0)	2 (33.3)
	Never married, divorced, or widowed	8 (80.0)	4 (100.0)	4 (66.7)
Cancer site	Breast	3 (30.0)	1 (25.0)	2 (33.3)
	Prostate	2 (20.0)	1 (25.0)	1 (16.7)
	Others	5 (50.0)	2 (50.0)	3 (50.0)

Note. * The chronological age difference between groups is not significant (z = −1.390, *p* = 0.171).

**Table 2:** Association between changes in DNA methylation age, symptoms, and resilience.

Variables	1	2	3	4	5	6	7
**1**. Δ DNA methylation age (Δ Age acceleration)	1	−0.145	0.235	0.684	0.493	−0.377	−0.897 *
**2**. Δ Pain		1	0.378	0.221	0.213	0.152	0.086
**3**. Δ Fatigue			1	0.39	0.027	0.119	−0.021
**4**. Δ Cognitive dysfunction				1	−0.54	−0.268	0.356
**5**. Δ Perceived cognitive difficulty					1	0.165	−0.572
**6**. Δ Insomnia						1	0.156
**7**. Δ Resilience							1

Note. * *p* < 0.05.

**Table 3:** DNA methylation age, symptoms, and resilience changes.

Measures	Control (*n* = 4)			Intervention (*n* = 6)			*p*
	T1 (Pre)	T2 (Post)	Mean Difference	T1 (Pre)	T2 (Post)	Mean Difference	
DNA methylation age (years) *	74.90 ± 1.11	75.47 ± 0.32	0.57 ± 0.80	66.77 ± 7.68	65.93 ± 8.94	−0.83 ± 1.26	0.200
Age acceleration (years) *	−2.10 ± 1.91	−1.47 ± 1.89	0.57 ± 0.80	2.10 ± 4.79	1.27 ± 4.02	−0.83 ± 1.26	0.200
Pain	45.68 ± 6.51	52.03 ± 10.63	6.35 ± 5.55	53.42 ± 11.88	51.60 ± 10.93	−1.82 ± 11.68	0.257
Fatigue	44.08 ± 7.63	48.78 ± 3.94	4.70 ± 10.58	50.80 ± 5.21	44.93 ± 6.55	−5.86 ± 8.00	0.067
Cognitive decline	23.50 ± 5.20	24.25 ± 4.50	0.75 ± 1.50	26.75 ± 3.20	24.50 ± 2.08	−2.25 ± 3.30	0.200
Perceived cognitive difficulty	82.75 ± 17.63	85.25 ± 14.66	2.50 ± 5.20	70.83 ± 22.60	67.83 ± 22.75	−3.00 ± 17.54	0.914
Insomnia	15.75 ± 3.86	14.50 ± 7.59	−1.25 ± 4.79	17.33 ± 6.25	13.33 ± 4.76	−4.00 ± 6.78	0.762
Resilience	34.50 ± 4.80	35.00 ± 4.00	0.50 ± 2.08	33.33 ± 11.04	36.17 ± 5.46	2.83 ± 6.43	0.914

Note. * *n* (control = 3, intervention = 3).

## Data Availability

The data presented in this study are available on request from the corresponding author upon reasonable request.
